# Effect of the local administration of betamethasone on pain, swelling and trismus after impacted lower third molar extraction. A randomized, triple blinded, controlled trial

**DOI:** 10.4317/medoral.19280

**Published:** 2013-10-13

**Authors:** José Marques, Jordi Pié-Sánchez, Rui Figueiredo, Eduard Valmaseda-Castellón, Cosme Gay-Escoda

**Affiliations:** 1 DDS. Master degree in Oral Surgery and Implantology. School of Dentistry, University of Barcelona. Barcelona, Spain; 2 DDS, MS, PhD. Master degree in Oral Surgery and Implantology. Professor of the Oral Surgery and Implantology Department. School of Dentistry, University of Barcelona. Researcher of the IDIBELL Institute. Barcelona, Spain; 3 DDS, MS, PhD. Master degree in Oral Surgery and Implantology. Professor of the Oral Surgery and Implantology Department. School of Dentistry, University of Barcelona. Researcher of the IDIBELL Institute. Barcelona, Spain; 4 MD, DDS, MS, PhD. Chairman and Full Professor of Oral and Maxillofacial Surgery. Director of the Master of Oral Surgery and Implantology. School of Dentistry, University of Barcelona. Coordinator Researcher of the IDIBELL Institute. Head of the Department of Oral and Maxillofacial Surgery, Teknon Medical Center. Barcelona, Spain

## Abstract

Objectives: The aim of this study is to compare the analgesic and anti-inflammatory effects of the local postoperative administration of a single 12-mg dose of betamethasone after the surgical removal of impacted lower third molars. Study Design: A split-mouth, triple-blind, randomized, placebo-controlled clinical trial of 25 patients requiring the surgical removal of symmetrical lower third molars was performed. In the experimental side, a 12-mg dose of betamethasone was administered submucosally after the surgical procedure, while in the control side a placebo (sterile saline solution) was injected in the same area. To assess postoperative pain, visual analogue scales and the consumption of rescue analgesic were used. The facial swelling and trismus were evaluated by measuring facial reference distances and maximum mouth opening. Results: There were no significant differences between the two study groups regarding postoperative pain, facial swelling and trismus. Conclusions: The injection of a single dose of betamethasone does not seem to reduce pain, facial swelling and trismus after impacted lower third molar removal when compared to placebo.

** Key words:**Third molar extraction, corticosteroids, betamethasone.

## Introduction

The most common postoperative complications of impacted lower third molar extractions are pain, trismus and facial swelling (1).Many papers have suggested several measures to prevent and treat these complications. Among these, the administration of non-steroidal anti-inflamatory drugs (NSAID) is considered to be one of the most useful (2,3). Nevertheless, some reports have showed that, a significant percentage of patients need additional medication to adequately control pain and swelling after this surgical procedure (2,3).

The intraoperative administration of corticosteroids is a pharmacologic approach that allows a reduction of the postoperative morbidity by inhibiting the synthesis and/or release of pro-inflammatory and inflammatory mediators in a variety of surgical procedures, with a reduction of fluid transudation and therefore edema (4). However, the prolonged use of corticosteroids can delay healing, increase patient susceptibility to infection and may cause adrenal suppression (5).

 The absolute contraindications to corticosteroid use include patients with tuberculosis, active viral or fungal infections, active acne vulgaris, primary glaucoma, history of acute psychosis or psychopathic tendencies and allergies (6). Since these contraindications refer to chronic corticosteroid use, such drugs should be avoided in patients with these problems (7).

A recent systematic review has concluded that there is no clear practice consensus concerning the use of corticosteroids in third molar removal because published studies lack comparability with regard to patient selection, dosage, timing, type, and route of administration of steroid (8).

Celestone Cronodose® (Schering-Plough S.A.; Madrid, Spain) is an injectable solution that contains two betamethasone esters, one of high solubility and other with a slow absorption, achieving a strong anti-inflammatory, anti-rheumatic and antiallergic effect. This preparation provides an immediate therapeutic effect due to betamethasone disodium phosphate (6 mg), a fast-acting soluble ester. The prolonged effect is due to betamethasone acetate (6 mg), which is slowly and gradually absorbed. This way, with a single injection, a rapid, prolonged and uniform steroid effect can be obtained (9). Futhermore, the selected corticosteroid should have scant mineralocorticoid effects and great biological activity.

The aim of this clinical trial was to evaluate the effect of the submucosal injection of a single 12-mg dose of betamethasone following the surgical extraction of impacted lower third molars under local anesthesia on pain, facial swelling and trismus.

## Patient and Methods

A randomized, triple-blind, split mouth, placebo-controlled clinical trial was performed in 25 patients. All participants were submitted to the surgical removal of both impacted lower third molars between the May 2008 and June 2010. This trial was design complying with the CONSORT guidelines for clinical trials (10).

The study was approved by the Research Ethics Committee (CEIC) of the Dental Clinic of the University of Barcelona. Before enrolment, all patients were explained the objectives, implications and possible complications of this clinical trial and agreed to participate by signing an informed consent. The main inclusion criterion was the presence of symmetrical impacted lower third molars that required surgical removal.

Exclusion criteria were patients aged below 18 years or over 35 years, patients with significant systemic diseases (ASA III or ASA IV), pregnancy, contraindications for corticosteroid treatment, history of allergy to paracetamol or magnesium metamizol, lactose intolerance, gastrointestinal pathology, presence of symptoms associated to the third molar the week prior to extraction and history of analgesic and/or anti-inflammatory drugs intake 10 days before. Antibiotic prophylaxis was not performed. Sequentially numbered envelopes were used to warrant allocation concealment.

The extractions were all of similar technical difficulty, and a panoramic radiography showed positioning of the teeth to be symmetrical (regarding Pell & Gregory and Winter classifications). All patients included required bone removal and tooth sectioning to achieve extraction. Informed consent was obtained in all cases for both surgical extraction and inclusion in the study.

The extractions were carried out by two third-year residents of the Master degree program of Oral Surgery and Implantology (University of Barcelona) using a similar surgical technique. Each resident performed both extractions on the same patient, with a washout period of 1 month.

The extraction of impacted lower third molars was performed under local anesthesia with articaine 4% and epinephrine 1:100.000 (Artinibsa; Inibsa, Lliça de Vall, Spain). The surgical field and all the surgical material were sterile. The surgeon raised a full-thickness flap, which was protected by the Minnesota retractor. A lingual flap retraction using a Freer periosteal elevator was only performed when the surgeon consider it to be necessary. Sterile low-speed (20.000 rpm) handpieces and sterile saline solution were used for bone removal and tooth sectioning when necessary. To close the wound, 3-0 silk sutures (Silkam, Braun; Tuttlingen, Germany) were used. The surgical technique was similar to that described by Leonard (11). The duration of surgery was calculated from the time of incision to placement of the last suture. The duration of the shortest intervention was required to be 75–100% that of the longest intervention.

No medication was given before extraction. The following medication was prescribed: an antibiotic (amoxicillin 750 mg, tablets (Clamoxyl®, GlaxoSmithKline, Madrid, Spain) p.o every 8 hours for 7 days), an analgesic (paracetamol 1 g, tablets (Gelocatil®, Gelos® SL; Barcelona, Spain) p.o every 8 hours for 5 days), a mouthrinse (0.12% chlorhexidine mouthwash 2 times a day for 15 days) and a rescue medication (magnesium metamizol 575 mg, tablets (Nolotil®, Boehringer Ingelheim España S.A.; Sant Cugat, Spain) 2 capsules p.o every 8 hours in case of pain). All patients were told not to apply ice during the postoperative period, in order to avoid possible bias.

The injection of corticosteroids in the right or left side was chosen randomly, using a predetermined sequence of random numbers in blocks (generated in www.randomization.com).

Following removal of the third molar in the corticosteroidal group, 12 mg of betamethasone (Celestone Cronodose®, Schering-Plough S.A.; Madrid, Spain) was injected submucosally through an intraoral approach. The control group received a sterile saline solution. The injection of the drug was carried out by a third person (not directly involved in the surgery nor in the postoperative visits), thus ensuring blinding of the study. The patients rated pain on a 10-cm visual analog scale, the extreme scores being ‘no pain’ and ‘worst pain imaginable’. Pain was assessed 6 hours after the end of surgical procedure, and then once a day during the next 3 days. The patients were also instructed to register the total amount of rescue medication needed every day and possible adverse events.

Facial swelling and trismus were registered at 48 hours and 7 days after the extraction by a blinded surgeon. Trismus was assessed by measuring the maximum mouth opening with a calliper, and facial swelling was given by the following facial distances: gonion-lip commissure, gonion-external canthus of the eye, tragus-lip commissure (12). The following variables were also gathered: Age, gender, smoking habit, position of the third molar (Pell and Gregory and Winter classifications), bone retention, bone removal and tooth sectioning.

All patients, the statistician and the surgeons who performed the extraction and follow-up examinations were unaware of the medication given in each extraction. The sample size was calculated using the statistical program G * Power 3.0. (Heinrich-Heine-Universität, Düsseldorf, Germany)8, with an alpha value of 0.05, a statistical power of 80%, and in order to de-tect differences of 4 mm in gonion-lip commissure distance.

The data obtained was analyzed using SPSS 19.0 statistical package (SPSS Inc., Chicago, IL, USA) for Microsoft Windows. Where distribution was compatible with normality, the mean and standard deviation (SD) were used. Pain, trismus, and facial edema were analyzed by ANOVA for a repeated-measures test. Rescue medication was assessed by Student t-tests. The significance level was set at p < 0.05 with a confidence interval of 95%.

## Results

**Figure 1:**
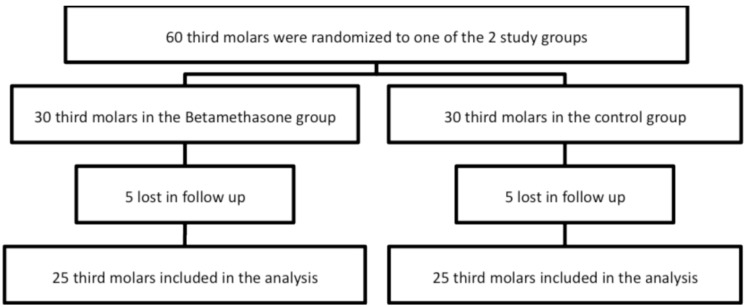
CONSORT flow chart of the participants in the trial.

A total of 30 patients were enrolled, although 5 were lost because they did not attend follow-up visits. Figure 1 shows a flow chart of the recruitment of participants 16. Therefore the results were based in the analysis of a total of 50 mandibular third molars extractions (25 participants), 25 in the experimental group and 25 in the control group as each patient act as its own control. The study groups were similar regarding bone retention of the third molar and duration of surgery (t-student tests; p>0.05). The main clinical variables of the sample can be observed in  Table 1.

**Table 1:**
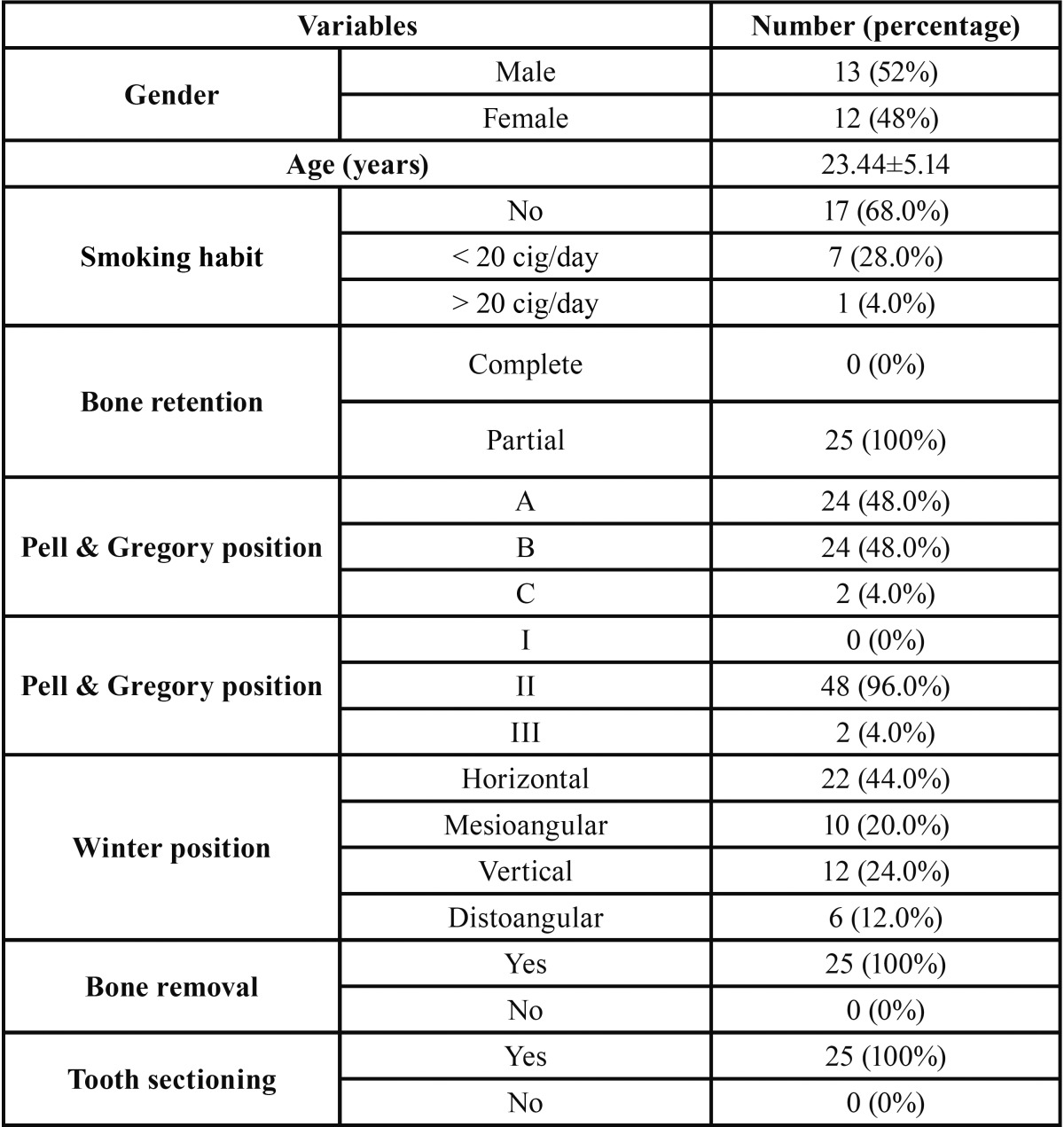
Main clinical features of the patients.

 Table 2 shows the results for pain intensity, rescue medication intake, facial swelling and mouth opening variables in the 2 study groups. The peak pain occurred at 6 hours and there were no significant differences between the two groups regarding pain intensity and rescue medication intake in the first 3 postoperative days (ANOVA repeated measures and t-student tests; p>0.05). When the analysis was made for each individual pain assessment time, once again no significant differences were found (t-student tests; p>0.05).

**Table 2:**
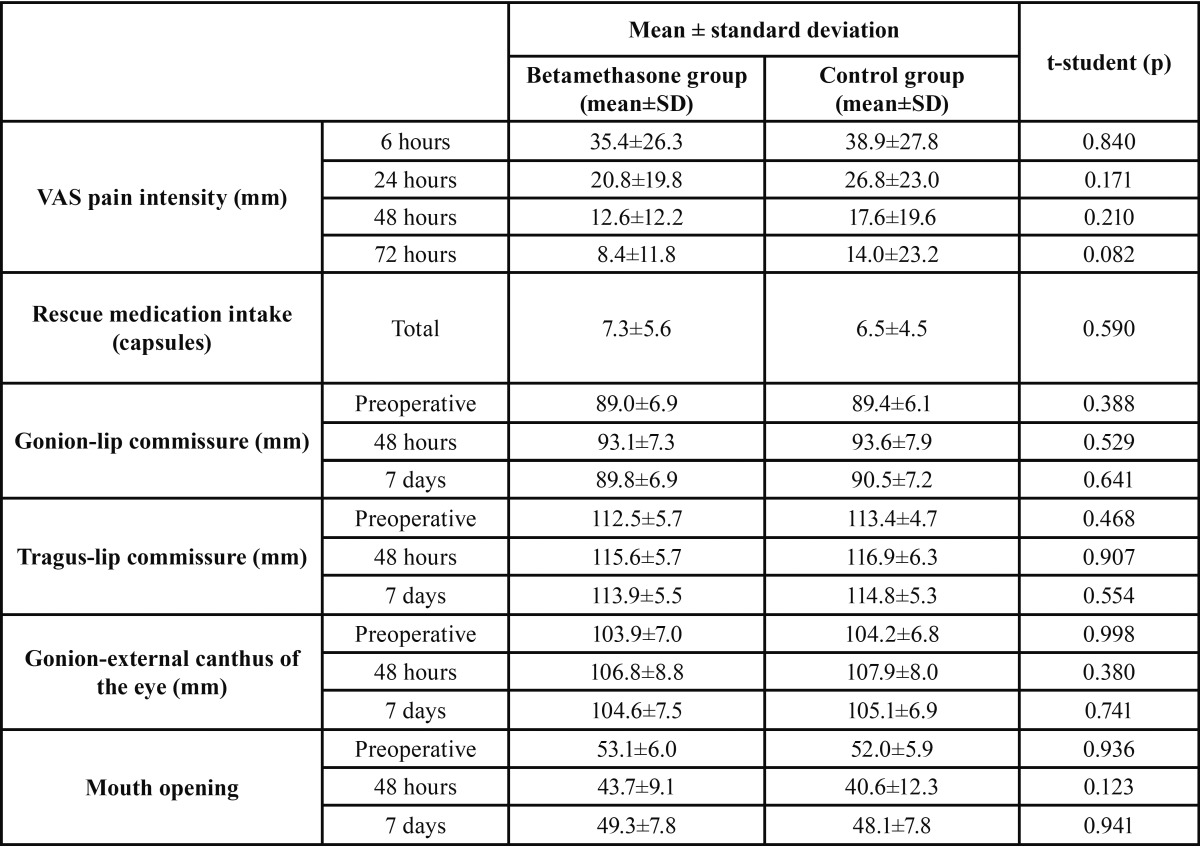
Comparison between the 2 study groups for postoperative pain, rescue medication intake, mouth opening and facial swelling.

The reference distances gonion-lip commissure, gonion-external canthus of the eye, tragus-lip commissure, and mouth opening were similar in both groups (ANOVA repeated measures and t-student tests; p>0.05).

No adverse effects related to the experimental medication or to any of the prescribed drugs were registered.

## Discussion

The third molar surgical extraction is often related with severe postoperative discomfort. Thus, many clinicians routinely use corticosteroids in order to prevent and reduce the postsurgical sequelae (4).

Several papers have shown a significant reduction of trismus, pain and facial swelling when corticosteroids are administered, but few reports use the local injection of these drugs in the third molar region (8,13) .

Different administration routes have been used for these drugs in oral surgery. The oral route is more comfortable for the patient and ensures rapid and almost complete absorption, but its efficacy compared with parenteral administration is questionable (6). The intravenous administration affords excellent and immediate plasma drug levels, although this route is not frequently used in an outpatient environment. Some studies show that a single preoperative intravenous dose offers almost immediate benefit in terms of pain, swelling and trismus, but frequently a supplemental dosing is needed, in order to ensure optimum clinical efficacy (6,14). With the present corticosteroid it was expected a significant and sustained anti-inflammatory effects.

The studies conducted to date involved low doses and brief periods of observation (6,8). The intramuscular route affords good plasma drug concentrations and prolonged anti-inflammatory action with a single pre- or postoperative dose (15).

The local administration of the corticosteroid is convenient for the surgeon, since the injection is carried out in close to the surgical area, and also for the patient, since the injection is painsless and it does not depend on the patient compliance (1,12,16).

Antunes et al. (17) and Boonsiriseth et al. (18) compared the effect of two routes of administration of 8 mg of dexamethasone on pain, trismus and edema in impacted third molar surgery, obtaining effective and similar results.

To our knowledge, it has not been published a single study that uses this corticosteroid association in the management of pain, trismus and swelling after third molar surgery. The present study is a unique, prospective, randomized, triple-blind, split mouth, placebo-controlled clinical trial experimenting this association in surgery in order to determine its effectiveness in the reduction of postoperative discomfort.

Previously, Micó-Lorens et al. (15) injected 40 mg of methylprednisolone into the gluteal zone following the extraction of impacted third molars, and reported good results 2 days after the operation in terms of swelling, pain and trismus, but after 7 days the differences were no longer significant (15). Likewise, Grossi et al. (12) reported beneficial effects on facial edema 48 hours after surgery, when 2 different concentrations (4mg and 8mg) of dexamethasone sodium phosphate were administered submucosally. However, no statistically significant effects were found regarding pain and trismus, and the results were quite similar for the 2 dosage regimens of dexamethasone (12).

Vegas-Bustamante et al. (1) demonstrated the efficacy of methylprednisolone, as a single 40mg dose, injected into the masseter muscle after the extraction of impacted lower third molars, in the reduction of swelling, trismus and pain.

Klongnoi et al. (16) alson reported that a single intramuscular injection of 8 mg dexamethasone can reduce postoperative facial swelling and pain, without affecting trismus after surgical extraction of impacted lower third molars.

Nevertheless, our results contrast with this studies, since no significant results were found in the reduction of postoperative discomfort. This could be a result of different factors: in the calculation of the sample size it was not taken in account the dropout rate which decreased the number of subjects or the flap elevation and tissue manipulation during the surgery that could have affected the concentration of the injected drug and impeded its absorption (17).

Another limitation was that only a single third molar was removed at each procedure, and the effect observed in the present study might be different for more extensive and lengthier procedures, as also referred Antunes et al.(17). Further studies, with a bigger sample size and a greater technical difficulty could show significant results.

The method used in the current study to measure facial swelling and trismus (calipers and silk thread) is valid, easy to use and inexpensive. However it is not the most sensitive method and could generate some bias. Other methodological approaches have also been described, such as clinical observation, subjective palpation, and the use of malleable metal rods, photographic techniques, or ultrasounds (14,19-21). Other authors like Esen et al. (14) also used a computed tomography to evaluate the facial edema which is, in our opinion, a highly reprehensible procedure, since it is being used an ionizing radiation only to evaluate the facial edema.

The results of this study indicate that there is no significant benefit of a single intramuscular dose of betamethasone over placebo concerning pain reduction, facial swelling and trismus after impacted lower third molar removal.
